# Supplementary material for: Point-of-care testing and treatment of sexually transmitted and genital infections during pregnancy in Papua New Guinea (WANTAIM trial): protocol for an economic evaluation alongside a cluster-randomised trial
Source: BMJ Open. 2021 Aug 12;11(8):e046308. doi: 10.1136/bmjopen-2020-046308 (PMC8362726; doi:10.1136/bmjopen-2020-046308)
Supplement: Author's manuscript [file bmjopen-2020-046308.draft_revisions.pdf]

BMJ Open

BMJ Open is committed to open peer review. As part of this commitment we make the peer review history of every article we publish publicly available.

When an article is published we post the peer reviewers' comments and the authors' responses online. We also post the versions of the paper that were used during peer review. These are the versions that the peer review comments apply to.

The versions of the paper that follow are the versions that were submitted during the peer review process. They are not the versions of record or the final published versions. They should not be cited or distributed as the published version of this manuscript.

BMJ Open is an open access journal and the full, final, typeset and author-corrected version of record of the manuscript is available on our site with no access controls, subscription charges or pay-per-view fees (http://bmjopen.bmj.com).

If you have any questions on BMJ Open's open peer review process please email info.bmjopen@bmj.com

BMJ Open

Protocol for an economic evaluation alongside a cluster-randomised trial of point-of-care testing and treatment of sexually transmitted and genital infections during pregnancy in Papua New Guinea (WANTAIM trial)

Journal:	*BMJ Open*
Manuscript ID	bmjopen-2020-046308
Article Type:	Protocol
Date Submitted by the Author:	26-Oct-2020

Complete List of Authors:	Batura, Neha; University College London, Institute for Global Health<br>saweri, olga; University of New South Wales, The Kirby Institute; Papua New Guinea Institute of Medical Research, Population Health and Demography<br>Vallely, Andrew; Papua New Guinea Institute of Medical Research, Sexual and Reproductive Health Unit; UNSW Sydney, The Kirby Institute<br>Pomat, Willie; PNG Institute of Medical Research, Infection and Immunity<br>Homer, Caroline ; Burnet Institute; University of Technology Sydney, Faculty of Health<br>Guy, Rebecca; The Kirby Instute, University of New South Wales, Sexual Health Program<br>Luchters, Stanley; Burnet Institute, Centre for International Health; Universiteit Gent<br>Mola, Glen ; School of Medicine and Health Sciences, University of Papua New Guinea<br>Vallely, Lisa; UNSW Australia, Kirby Institute -Faculty of Medicine<br>Morgan, Christopher; Burnet Institute,<br>Kariwiga, Grace; Milne Bay Provincial Health Authority<br>Wand, Handan; The Kirby Institute for Infection and Immunity in Society, University of New South Wales<br>Rogerson, Stephen; University of Melbourne, Doherty Institute, Department of Medicine<br>Tabrizi, Sepehr; The Royal Women's Hospital,<br>Whiley, David; The University of Queensland, Queensland Children's Medical Research Institute<br>Low, Nicola; University of Bern, Bern, Switzerland, Institute of Social and Preventive Medicine<br>Peeling, Rosanna; London School of Hygiene and Tropical Medicine, Clinical Research<br>Siba, Peter; The Papua New Guinea Institute of Medical Research, Sexual and Reproductive Health Unit<br>Riddell, Michaela; University of New South Wales Faculty of Science, Kirby Institute<br>Laman, M.; Papua New Guinea Institute of Medical Research,<br>Bolnga, John ; Papua New Guinea Institute of Medical Research; The Kirby Institute for infection and immunity in society<br>Robinson, Leanne; Papua New Guinea Institute of Medical Research; Burnet Institute

	Morewaya, Jacob; Milne Bay Provincial Health Authority<br>Badman, Steven; UNSW Australia, Kirby Insitute<br>Kelly-Hanku, Angela; Papua New Guinea Institute of Medical Research; The Kirby Institute<br>Toliman, Pamela ; Papua New Guinea Institute of Medical Research<br>Peter, Wilfred; Provincial Health Office<br>Peach, Elizabeth; Burnet Institute<br>Garland, Suzanne; The Royal Women's Hospital, Microbiology and Infectious Diseases Department<br>Kaldor, John; University of New South Wales, The Kirby Institute<br>Wiseman, Virginia; The Kirby Institute; London School of Hygiene & Tropical Medicine

Keywords:	HEALTH ECONOMICS, Public health < INFECTIOUS DISEASES, Protocols & guidelines < HEALTH SERVICES ADMINISTRATION & MANAGEMENT

SCHOLARONE™
Manuscripts

I, the Submitting Author has the right to grant and does grant on behalf of all authors of the Work (as defined in the below author licence), an exclusive licence and/or a non-exclusive licence for contributions from authors who are: i) UK Crown employees; ii) where BMJ has agreed a CC-BY licence shall apply, and/or iii) in accordance with the terms applicable for US Federal Government officers or employees acting as part of their official duties; on a worldwide, perpetual, irrevocable, royalty-free basis to BMJ Publishing Group Ltd ("BMJ") its licensees and where the relevant Journal is co-owned by BMJ to the co-owners of the Journal, to publish the Work in this journal and any other BMJ products and to exploit all rights, as set out in our licence.

The Submitting Author accepts and understands that any supply made under these terms is made by BMJ to the Submitting Author unless you are acting as an employee on behalf of your employer or a postgraduate student of an affiliated institution which is paying any applicable article publishing charge ("APC") for Open Access articles. Where the Submitting Author wishes to make the Work available on an Open Access basis (and intends to pay the relevant APC), the terms of reuse of such Open Access shall be governed by a Creative Commons licence – details of these licences and which Creative Commons licence will apply to this Work are set out in our licence referred to above.

Other than as permitted in any relevant BMJ Author's Self Archiving Policies, I confirm this Work has not been accepted for publication elsewhere, is not being considered for publication elsewhere and does not duplicate material already published. I confirm all authors consent to publication of this Work and authorise the granting of this licence.

For peer review only

Protocol for an economic evaluation alongside a cluster-randomised trial of point-of-care testing and treatment of sexually transmitted and genital infections during pregnancy in Papua New Guinea (WANTAIM trial)

Authors

Batura N, Saweri OPM, Vallely AJ, Pomat WS, Homer C, Guy R, Luchters S, Mola GDL, Vallely LM, Morgan C, Kariwiga G, Wand H, Rogerson S, Tabrizi S, Whiley DM, Low N, Peeling R, Siba P, Riddell MA, Laman M, Bolnga J, Robinson L, Morewaya J, Badman SG, Kelly-Hanku A, Toliman P, Peter W, Peach E, Garland SM, Kaldor JM, and Wiseman V

For peer review only

Abstract

Introduction: Left untreated, sexually transmitted and genital infections (henceforth STIs) in pregnancy can lead to serious adverse outcomes for mother and child. Papua New Guinea (PNG) has amongst the highest prevalence of curable STIs including syphilis, chlamydia, gonorrhoea, trichomoniasis and bacterial vaginosis, and high neonatal mortality rates. Diagnosis and treatment of these STIs in PNG relies upon syndromic management but advances in STI diagnostics through point-of-care (PoC) testing using GeneXpert technology holds promise for resource-constrained countries such as PNG. This paper describes the planned economic evaluation of a cluster-randomised crossover trial comparing antenatal PoC testing and immediate treatment of curable STIs with standard antenatal care in two provinces in PNG.

Methods and analysis: Cost-effectiveness of the PoC intervention compared with standard antenatal care will be assessed prospectively over the trial period (2017-2021) from societal and provider perspectives. Incremental cost-effectiveness ratios will be calculated for the primary health outcome, a composite measure of the proportion of either preterm birth and/or low birth weight; for life years saved and disability-adjusted life years (DALY) averted; and for non-health benefits (financial risk protection) and improved health equity. Scenario analyses will be conducted to identify different scale-up options, and budget impact analysis will be undertaken to understand the short-term financial impact of intervention adoption on the national budget. Deterministic and probabilistic sensitivity analysis will be conducted to account for uncertainty in key model inputs.

Ethics and dissemination: This study has ethical approval from the Institutional Review Board of the PNG Institute of Medical Research; the Medical Research Advisory Committee of the PNG National Department of Health; the Human Research Ethics Committee of the University of New South Wales; and the Research Ethics Committee of the London School of Hygiene and Tropical Medicine. Findings from this study will be disseminated through national stakeholder meetings, conferences, peer-reviewed publications and policy briefs.

Trial registration: ISRCTN37134032.

Key words

Cost effectiveness analysis, economic evaluation, budget impact, equity, sexually transmitted infections, point of care testing, Papua New Guinea

For peer review only

Strengths and limitations of this study

- This protocol will assist in designing economic evaluations for similar complex public health interventions especially those that seek to capture both health and non-health impacts of point-of-care testing for STIs in low– and middle-income countries.
- This protocol follows the Consolidated Health Economic Evaluation Reporting Standards and guidelines from the Global Health Cost Consortium to design and report economic evaluations nested in a randomised controlled trial and will include individual level patient cost and health service use data.
- The planned budget impact and affordability analyses will contribute to the understanding of fiscal space for investments in maternal and child health at provincial and country levels.
- This analysis builds on conventional cost-effectiveness analysis by including non-health benefits such as financial risk protection that are key criteria for equitable resource allocation and the design of health benefit packages in many low– and middle-income countries including PNG.
- The planned analyses for the base case scenario adopts only a 12-month time horizon but we propose to model the costs and benefits over the lifetime of mother and their babies using published data.

For peer review only

INTRODUCTION

In 2017, it was estimated that every day, globally, more than 1 million people acquire any of the four common curable sexually transmitted infections: chlamydia, gonorrhoea, syphilis, and trichomoniasis [1-3]. Left untreated, sexually transmitted and genital infections such as bacterial vaginosis (henceforth, referred to as STIs) are associated with adverse pregnancy and birth outcomes including spontaneous abortion, miscarriage, stillbirth, pre-term birth, low birth weight, postpartum endometritis, premature rupture of membranes, postpartum endometritis and various sequelae in newborn infants owing to mother-to-child transmission such as ophthalmia neonatorum [4-13].

In Papua New Guinea (PNG), prevalence of STIs are high in the general population and among pregnant women [14]. Clinical studies in PNG show that around 50% of all pregnant women test positive for one or more STIs at their first antenatal care (ANC) visit [15 16], with gonorrhoea, chlamydia, trichomoniasis, and bacterial vaginosis most commonly diagnosed [15-18]. There is evidence to suggest that increased screening for HIV and syphilis in pregnancy is correlated with a reduction in perinatal and infant morbidity and mortality [19 20]. In this high burden and resource constrained setting, poor access to ANC leads to missed opportunities for early diagnosis and clinical intervention [21].

Traditional STI diagnosis for infections other than HIV and syphilis relies on microscopy, culture, and/or serology that requires technical resources and expertise that may not be readily available in all low- and middle-income country (LMIC) settings [22 23]. The long waiting period for results also deters some people from returning to collect their results [24]. In settings where laboratory services are not available, syndromic management that uses clinical presentation is used to inform treatment decisions. This strategy fails to detect asymptomatic infections and consequently leads to negligible impact on health outcomes [24 25]. The development of accurate rapid diagnostic tests for HIV and syphilis used at point-of-care (PoC) has shown to improve detection, testing coverage and the number of patients accurately diagnosed and treated [26-28]. From an equity standpoint, PoC testing has been shown to improve access to testing and treatment particularly amongst remote and hard to reach populations [26 29]. However, the success of HIV and syphilis PoC detection programmes is yet to be replicated for other common curable STIs [30-34].

For peer review only

The Women and Newborn Trial of Antenatal Interventions and Management (WANTAIM) study is the first randomised trial to evaluate the effectiveness and cost-effectiveness of PoC STI testing and treatment to improve birth outcomes in high-burden settings (Trial registration number: ISRCTN37134032) [35]. The aim of this paper is to describe the rationale and methodological approach for the economic evaluation component of this large-scale trial involving 4,600 pregnant women in PNG.

In recent years, the evidence base for the cost and cost-effectiveness of PoC testing for STIs in pregnancy has grown, including in LMICs. A recent systematic review [36] identified that the bulk of these studies were conducted in Africa or Latin and South America [37-52], with no studies undertaken in East Asia or the Pacific. Most of the studies investigated the cost and cost-effectiveness of PoC testing for syphilis in pregnancy compared to no screening, syndromic management or onsite laboratory testing. Only one study evaluated testing for HIV and testing and treatment for syphilis [48], another for chlamydia [41], and none evaluated testing for gonorrhoea, trichomoniasis, or bacterial vaginosis. Few studies evaluated the costs and cost effectiveness of the test and treatment package together. Despite widespread acknowledgement of the high out-of-pocket costs incurred by women and their families in accessing testing and treatment in many LMICs, the studies in the review were largely conducted from the provider perspective [40 41 45 49 50 52-55]. Further, none of the studies presented estimates of affordability or budget impact and none analysed any non-health related outcomes such as equity or financial risk protection [36].

STUDY SETTING

Papua New Guinea

In PNG, pregnant women and their infants experience a high burden of adverse health outcomes with PNG recording one of the highest maternal and neonatal mortality ratios in the world: 594 per 100,000 and 25 per 1000 live births, respectively, compared with global figures of 209 and 18 [56 57]. In 2012, 20% of births in PNG were preterm birth and/or low birth weight, both key contributors to neonatal mortality [58].

Pregnant women in PNG experience a high burden of curable STIs. Findings from a country-wide bio-behavioural survey of STIs in pregnancy indicated that the prevalence of chlamydia was 23%, gonorrhoea 14%, and trichomoniasis 22%, with 44% of women having at least one of these infections [5]. Another study evaluating the feasibility of a novel PoC testing and treatment strategy for STIs in PNG found that 54% of women had one or more of chlamydia,

gonorrhoea, trichomoniasis, or bacterial vaginosis, and the prevalence of each of these STIs was 19%, 11%, 38%, and 18% respectively [22]. Similar prevalence rates of STIs were observed in a study of malaria prevention in pregnancy [6]. In these studies, between 65-80% of infections among pregnant women was asymptomatic indicating the need for more accurate diagnosis at the point -of -care.

In PNG, PoC testing and treatment for HIV and syphilis is available for all pregnant women at the first ANC clinic visit, and women who test positive for either are immediately treated according to national guidelines [59]. However, despite the high prevalence of chlamydia, gonorrhoea, trichomoniasis and bacterial vaginosis amongst pregnant women, detection and treatment relies on syndromic management according to national guidelines [59].

Health services in Papua New Guinea

In PNG, health services are organized into seven levels of care. Levels 1-4 offer primary care at community health posts, health centres, sub-health centres and rural/district hospitals. The majority of Level 1-3 facilities are managed and staffed by health extension officers (HEOs), Nursing Officers (NOs) and community health workers (CHW); Level 4 facilities i.e. district/rural hospitals usually have a doctor on staff. Population coverage varies from about 5,000 to 20,000 per facility and the average distance travelled to reach a facility is 7-8 km. Secondary level care is provided at provincial/regional/national referral hospitals (Levels 5-7), which cover an average population of 200,000 and 300,000 in one or more provinces [60 61]. Health workforce distribution is suboptimal, with 0.5 physicians per 10,000 population [62], compared to the World Health Organization (WHO) recommended ratio of 10 physicians per 10,000 population [63]. Healthcare is predominantly provided by public health facilities that are either financed and operated by the PNG government or by churches with financial support from the government [64].

The WANTAIM trial

WANTAIM aims to test the effectiveness of antenatal PoC testing and treatment for STIs to improve maternal and newborn outcomes in PNG. WANTAIM is being implemented in two provinces in PNG – Madang and East New Britain Province. Data collection is underway and the trial is due to finish in late 2021 [35].

WANTAIM is a cluster-randomised crossover trial and the unit of randomization is a primary health care centre and its catchment area. Ten geographically distinct clusters have been

For peer review only

assigned in a 1:1 ratio to intervention and control arms in the first phase of the trial. The end of the first phase of the trial is followed by a short washout period of two to three months, at the end of which each cluster will cross over to participate in the alternative trial arm in the second phase. The study participants are women attending their first ANC visit, aged over 16 years and less than 26 weeks' gestation (assessed by ultrasound) (n=4,600). Newborn infants are followed up within 72 hours of birth.

Pregnant women recruited into the study receive routine ANC as per PNG national guidelines including sulphadoxine-pyrimethamine (SP) for malaria prevention; iron and folate supplementation; tetanus toxoid immunization; HIV and syphilis screening (and treatment if required). Women in the control arm receive STI syndromic management if they report symptoms of genital infection (abdominal pain, discharge). They also provide a urine sample for diagnostic testing in the study laboratory. If positive for STI at their last test, they receive treatment during the first postnatal visit. Women in the intervention arm of the trial provide a self-collected vaginal specimen for PoC STI testing, and same day treatment as necessary, at the following time points:

- At enrolment (<26 weeks gestation)
- One month after trial enrolment; and
- At 34-36 weeks antenatal follow-up.

The primary outcome of the trial is a composite measure of two events, the proportion of women and their newborn infants in each trial arm who experience either a preterm birth (<37 weeks gestation) and/or low birth weight (<2500g).

The study has ethical approval from the Institutional Review Board of the Papua New Guinea (PNG) Institute of Medical Research (IRB number 1608); the Medical Research Advisory Committee of the PNG National Department of Health (MRAC number 16.24); the Human Research Ethics Committee of the University of New South Wales (HREC number 16708); and the Research Ethics Committee of the London School of Hygiene and Tropical Medicine (REC number 12009).

A full description of the WANTAIM intervention and trial design is described elsewhere [35]. The purpose of this paper is to fully describe the methods for the economic evaluation of the trial.

For peer review only

AIMS AND OBJECTIVES

The aim of the economic evaluation is to assess the cost-effectiveness and affordability of PoC testing and treatment of curable STIs in pregnancy compared to standard care from a provider and societal perspective. The specific objectives of the economic evaluation are to:

1. Estimate total financial and economic costs of the PoC STI intervention;
2. Model incremental cost-effectiveness of the intervention compared to standard care;
3. Extend the incremental cost-effectiveness analysis to include equity related measures of impact; and
4. Conduct a budget impact analysis to assess the affordability of implementing the intervention at the national level or in target areas/populations.

These planned analyses will adhere to the Consolidated Health Economic Evaluation Reporting Standards and established guidelines from the Global Health Cost Consortium for conducting and reporting economic evaluation for global health trials [65 66].

METHODS: ECONOMIC EVALUATION OF THE WANTAIM TRIAL

Costing data

Cost data collection is guided by the perspective adopted for the economic evaluation. For WANTAIM, direct and indirect costs will be collected from the provider perspective and societal perspective – the latter including any costs incurred by pregnant women and their families. The different cost categories and data sources are summarised in Table 1. A combination of top-down and bottom-up costing approaches will be used [65] and the time horizon for the main trial-based economic evaluation will be 12 months.

Table 1: Cost category, and data sources

**Description**	**Type of cost**	**Data sources**	**Sample size**
Provider costs			
Costs of implementing WANTAIM	Direct	Project accounts of implementing agencies	N/A
Cost of providing ANC services	Direct	Health facilities	10 health facilities
Cost of increased workload of facility staff associated with PoC testing and treatment	Indirect	Patient pathway and health worker observation data collected as part of the health facility assessment	20-30
Participant costs			
Costs of care seeking	Direct and indirect	Participant case report forms	4,600

Provider costs are incurred by the institutions implementing the PoC testing intervention across the start-up, implementation and monitoring phases of the trial. The cost data will be sourced from financial records, programme documents and consultation with project staff. A step-down costing methodology will be used whereby costs from project accounts are entered into a customized tool created in Microsoft Excel, which is adapted each year to reflect the changing cost structure of the trial during the start-up and implementation phases.

Financial costs will be converted to economic costs i.e. any donated goods or volunteer time that do not appear in the programme accounting data will be added to the cost sheets and assigned a current market value [67 68]. Key informant interviews with programme leads will assist in identifying donated or subsidised items and in allocating joint costs between programme components. The allocation of joint staff costs is informed by monthly staff time sheets. Research costs will not be included in the cost-effectiveness analysis. However, start-up costs will be reported and differentiated from implementation costs to enable decision-makers to gauge the costs associated with the initial activities and expenditures necessary to develop PoC testing and integration with standard ANC [69].

Provider (treatment) costs are incurred by Provincial Health Authorities, who manage ANC, delivery, and postnatal visits, and Church Health Services; non-state providers who access a mix of government and institutional funds. Primary data on the average unit cost of care will be collected from all health facilities participating in the WANTAIM trial. A simple cost-capture form has been developed for facility data collection adapted from other costing studies led by members of this team [70 71]. Data from this form will be used to complement existing data from centre reports, patients' records and published national reports relating to ANC, labour and birth care, and PNC visits. Costs of services provided will also be calculated using a step-down approach. [72]

Participant (treatment) costs are the direct and indirect costs of health care seeking incurred by women and their families such as medical costs, transport costs, and the opportunity costs in terms of lost productivity due to care seeking visits. These will be estimated for standard treatment episodes in the control arms and for treatment episodes in the intervention arm to gauge changes in out-of-pocket costs of care-seeking and time dedicated to care-seeking for participants. Data on the direct and indirect costs incurred by participants are being collected from all trial participants (n=4600) in both arms of the trial at enrolment and three follow up

For peer review only

visits through participant case report forms (CRFs).

Health service use

Health service utilization for all trial participants in the intervention and control arms will be estimated using data collected via a take-home aide memoire and participant CRFs. The aide memoire is provided to all participants at recruitment, who use this tool to make notes about the facility visits that they make or attend between the WANTAIM follow-up visits. The aide memoire also allows them to make notes about any costs associated with those visits. At the WANTAIM follow-up visits, these notes serve as prompts for questions about service utilization and costs of careseeking, which are recorded in the CRFs.

Proposed analyses

Cost and cost effectiveness analyses:

A base-case analysis will be undertaken alongside the trial to estimate the cost-effectiveness of the intervention compared with standard care as implemented. The base case will include all start-up costs and implementation costs. Costs will be presented in current prices in PNG Kina and International Dollars. All costs will be adjusted for inflation using the Consumer Price Index (CPI) for PNG and will be converted to 2021 International Dollars (INT$) using the 2021 Purchasing Power Parity (PPP) conversion factor for PNG. Costs and outcomes will be converted to present values using an annual discount rate of 3% in the base-case, and annual rates of 0%, 6% and 9% in sensitivity analyses.

For base case analysis, results will be presented in terms of total financial and economic costs of the intervention and incremental cost-effectiveness ratios (ICERs) for the primary outcome i.e. the proportion of women and their newborns who experience either preterm birth and/or low birth weight. ICERs will be calculated as the arithmetic mean difference in cost between the intervention and control arms, divided by the arithmetic mean difference in effect. In order to maximise comparability with other trials, ICERs will also be reported in terms of cost per life year saved and cost per disability adjusted life year (DALY) averted (see 'Modelling' section for details).

The data on costs and outcomes for the period of trial follow-up will be at the individual-level, allowing evaluation of uncertainty of the cost-effectiveness estimates using non-parametric bootstrapping [73]. Cost effectiveness acceptability curves (CACs) will be generated to further describe uncertainty around the cost estimates [74]. CACs indicate the proportion of the

For peer review only

estimates produced by bootstrapping that would be 'acceptable' below a range of willingness to pay thresholds, where willingness to pay is the value placed on an additional pregnant woman appropriately tested for STIs in pregnancy. Sensitivity analyses will take into account the uncertainty in key parameters that may have been affected by the COVID-19 pandemic, such as staff or drugs costs.

Modelling:

The base case analysis will have a time horizon of up to 12 months. If the intervention demonstrates clinical effectiveness over that period, we will employ decision analytic modelling to examine the cost-effectiveness of the intervention over a newborn's lifetime. The model will employ Markov-type of long-term health benefits, healthcare costs and cost-effectiveness of the PoC intervention compared to standard care, drawing on results of the WANTAIM trial and available published data. The model will be used to project differences between the intervention and control arms in life years saved, DALYs averted, and lifetime healthcare costs. Sensitivity analyses will also be conducted within this model.

Equity impact of the intervention:

The equity impact of the intervention will be investigated by conducting an extended cost-effectiveness analysis (E-CEA). The E-CEA broadens the scope of the CEA by incorporating health equity and financial protection considerations for the most vulnerable sections of the population, that are likely to have the highest need [75]. This will be done across three domains: by exploring improving health gains, with particular reference to the poorest socio-economic group; reduction in the out-of-pocket expenses faced by households seeking care; and improved financial protection or reducing the number of households that sink into poverty due to catastrophic health spending [76]. For the E-CEA, provider and participant cost data (Table 1) will be synthesised with data on service utilization that will be collected via participant CRFs that are completed at enrolment into the trial and at three follow up trial visits. All results will be presented by socio-economic quintiles. Given that socio-economic groups may not differ greatly within clusters, a multidimensional poverty index (MDPI) will be derived from socio-economic and income data collected from all trial participants at enrolment. The use of an MDPI provides a more nuanced understanding of socioeconomic status of households as it takes monetary and non-monetary dimensions of deprivation into account [77]. This enables the differentiation between population groups who may all be relatively poor in monetary dimensions such as income or asset ownership [78] Thus, the

For peer review only

consideration of other non-monetary attributes (e.g. housing) allows us to distinguish between households that are homogenously asset or cash poor in this study setting [78].

Scale-up and budget impact analysis:

The costs and cost-effectiveness of the intervention will also be considered in a scale-up scenario, in which any start-up costs will be excluded as they are considered sunk costs [79]. The budget impact of the scale up scenario will be explored by an analysis of fiscal space for programme delivery using a generalised fiscal space assessment method [80 81] and probabilistic analyses to determine a set of cost-effectiveness thresholds [74 82].

DISCUSSION

To our knowledge, this paper is the first protocol for the economic evaluation of PoC STI testing and treatment in pregnancy in a LMIC setting. The proposed analyses aim to assess the cost-effectiveness of the intervention as well as its affordability and equity impact. The analyses will adhere to international guidelines for conducting and reporting economic evaluation studies and provide transparency in how they are conducted. The findings of the economic evaluation will provide decision-makers in PNG and similar settings evidence on the relative value for money of this intervention and the likely level of investment required for implementation at scale. The findings of this study will be disseminated through national stakeholder meetings, conferences, per-reviewed publications and policy briefs.

PATIENT AND PUBLIC INVOLVEMENT

Patients and public are not involved in the process of the economic evaluation of the WANTAIM trial.

DISSEMINATION

The findings of the economic evaluation of the WANTAIM trial will be disseminated to academic and policy-making communities, and the wider public, in a peer-reviewed journals and presented at a relevant conferences in Papua New Guinea and globally.

For peer review only

REFERENCES

1. Newman L, Rowley J, Vander Hoorn S, et al. Global estimates of the prevalence and incidence of four curable sexually transmitted infections in 2012 based on systematic review and global reporting. *PloS one* 2015;10(12):e0143304.
2. World Health Organization. Sexually transmitted infections: implementing the global STI strategy: World Health Organization, 2017.
3. Unemo M, Bradshaw CS, Hocking JS, et al. Sexually transmitted infections: challenges ahead. *The Lancet infectious diseases* 2017;17(8):e235-e79.
4. Mullick S, Watson-Jones D, Beksinska M, et al. Sexually transmitted infections in pregnancy: prevalence, impact on pregnancy outcomes, and approach to treatment in developing countries. *Sexually transmitted infections* 2005;81(4):294-302.
5. Vallely LM, Toliman P, Ryan C, et al. Prevalence and risk factors of Chlamydia trachomatis, Neisseria gonorrhoeae, Trichomonas vaginalis and other sexually transmissible infections among women attending antenatal clinics in three provinces in Papua New Guinea: a cross-sectional survey. *Sexual health* 2016;13(5):420-27.
6. Wangnapi R, Soso S, Unger H, et al. Prevalence and risk factors for Chlamydia trachomatis, Neisseria gonorrhoeae and Trichomonas vaginalis infection in pregnant women in Papua New Guinea. *Sex Transm Infect* 2015;91(3):194-200.
7. Gravett MG, Nelson HP, DeRouen T, et al. Independent associations of bacterial vaginosis and Chlamydia trachomatis infection with adverse pregnancy outcome. *Jama* 1986;256(14):1899-903.
8. John Hopkins Study Investigators. Association of Chlamydia trachomatis and Mycoplasma hominis with intrauterine growth retardation and preterm delivery. The John Hopkins Study of Cervicitis and Adverse Pregnancy Outcome. *Am J Epidemiol* 1989;129(6):1247-57.
9. Elliott B, Brunham RC, Laga M, et al. Maternal gonococcal infection as a preventable risk factor for low birth weight. *Journal of infectious diseases* 1990;161(3):531-36.
10. de Attayde MJPM, Florêncio GLD, Gabiatti JRE, et al. Perinatal morbidity and mortality associated with chlamydial infection: a meta-analysis study. *The Brazilian Journal of Infectious Diseases* 2011;15(6):533-39.
11. Rours GIJ, Duijts L, Moll HA, et al. Chlamydia trachomatis infection during pregnancy associated with preterm delivery: a population-based prospective cohort study. *European journal of epidemiology* 2011;26(6):493-502.
12. Johnson HL, Ghanem KG, Zenilman JM, et al. Sexually transmitted infections and adverse pregnancy outcomes among women attending inner city public sexually transmitted diseases clinics. *Sexually transmitted diseases* 2011;38(3):167-71.
13. Kahn JG, Jiwani A, Gomez GB, et al. The cost and cost-effectiveness of scaling up screening and treatment of syphilis in pregnancy: a model. *PLoS One* 2014;9(1)
14. Blencowe H, Cousens S, Jassir FB, et al. National, regional, and worldwide estimates of stillbirth rates in 2015, with trends from 2000: a systematic analysis. *The Lancet Global Health* 2016;4(2):e98-e108.
15. Badman SG, Vallely LM, Toliman P, et al. A novel point-of-care testing strategy for sexually transmitted infections among pregnant women in high-burden settings: results of a feasibility study in Papua New Guinea. *BMC infectious diseases* 2016;16:250-50. doi: 10.1186/s12879-016-1573-4
16. Wangnapi RA, Soso S, Unger HW, et al. Prevalence and risk factors for Chlamydia trachomatis, Neisseria gonorrhoeae and Trichomonas vaginalis infection in pregnant women in Papua New Guinea. *Sexually Transmitted Infections* 2015;91(3):194-200. doi: 10.1136/sextrans-2014-051670

For peer review only - http://bmjopen.bmj.com/site/about/guidelines.xhtml

17. Vallely A, Page A, Dias S, et al. The prevalence of sexually transmitted infections in Papua New Guinea: a systematic review and meta-analysis. *PloS one* 2010;5(12):e15586-e86. doi: 10.1371/journal.pone.0015586
18. Vallely LM, Toliman P, Ryan C, et al. Prevalence and risk factors of Chlamydia trachomatis, Neisseria gonorrhoeae, Trichomonas vaginalis and other sexually transmissible infections among women attending antenatal clinics in three provinces in Papua New Guinea: a cross-sectional survey. *Sexual Health* 2016;13(5):420-27. doi: https://doi.org/10.1071/SH15227
19. Watson-Jones D, Oliff M, Terris-Prestholt F, et al. Antenatal syphilis screening in sub-Saharan Africa: lessons learned from Tanzania. *Tropical Medicine & International Health* 2005;10(9):934-43. doi: 10.1111/j.1365-3156.2005.01473.x
20. Blencowe H, Cousens S, Kamb M, et al. Lives Saved Tool supplement detection and treatment of syphilis in pregnancy to reduce syphilis related stillbirths and neonatal mortality. *BMC public health* 2011;11(S3):S9.
21. Ministerial Task Force on Maternal Health in Papua New Guinea. Report of the Ministerial Task Force on Maternal Health in Papua New Guinea. . Port Moresby, Papua New Guinea: National Department of Health, Government of Papua New Guinea, 2009.
22. Badman SG, Vallely LM, Toliman P, et al. A novel point-of-care testing strategy for sexually transmitted infections among pregnant women in high-burden settings: results of a feasibility study in Papua New Guinea. *BMC infectious diseases* 2016;16(1):250.
23. Toskin I, Blondeel K, Peeling RW, et al. Advancing point of care diagnostics for the control and prevention of STIs: the way forward. *Sex Transm Infect* 2017;93(S4):S81-S88.
24. Vallely LM, Toliman P, Ryan C, et al. Performance of syndromic management for the detection and treatment of genital Chlamydia trachomatis, Neisseria gonorrhoeae and Trichomonas vaginalis among women attending antenatal, well woman and sexual health clinics in Papua New Guinea: a cross-sectional study. *BMJ open* 2017;7(12):e018630-e30. doi: 10.1136/bmjopen-2017-018630
25. Peeling RW. Testing for sexually transmitted infections: a brave new world? *Sexually Transmitted Infections* 2006;82(6):425-30. doi: 10.1136/sti.2005.017251
26. Mabey DC, Sollis KA, Kelly HA, et al. Point-of-Care Tests to Strengthen Health Systems and Save Newborn Lives: The Case of Syphilis. *PLOS Medicine* 2012;9(6):e1001233. doi: 10.1371/journal.pmed.1001233
27. Marks M, Mabey DC. The introduction of syphilis point of care tests in resource limited settings. *Expert review of molecular diagnostics* 2017;17(4):321-25. doi: 10.1080/14737159.2017.1303379 [published Online First: 2017/03/14]
28. Swartzendruber A, Steiner RJ, Adler MR, et al. Introduction of rapid syphilis testing in antenatal care: A systematic review of the impact on HIV and syphilis testing uptake and coverage. *International Journal of Gynaecology & Obstetrics* 2015;130 Suppl 1:S15-21.
29. Phang Romero Casas C, Martyn-St James M, Hamilton J, et al. Rapid diagnostic test for antenatal syphilis screening in low-income and middle-income countries: a systematic review and meta-analysis. *BMJ Open* 2018;8(2):e018132.
30. Nuñez-Forero L, Moyano-Ariza L, Gaitán-Duarte H, et al. Diagnostic accuracy of rapid tests for sexually transmitted infections in symptomatic women. *Sexually transmitted infections* 2016;92(1):24-28.
31. Pearce DM, Styles DN, Hardick JP, et al. A new rapid molecular point-of-care assay for Trichomonas vaginalis: preliminary performance data. *Sexually transmitted infections* 2013;89(6):495-97.

For peer review only

32. Sabido M, Hernandez G, Gonzalez V, et al. Clinic-based evaluation of a rapid point-of-care test for detection of Chlamydia trachomatis in specimens from sex workers in Escuintla, Guatemala. *Journal of clinical microbiology* 2009;47(2):475-76.
33. Hegazy MM, El-Tantawy NL, Soliman MM, et al. Performance of rapid immunochromatographic assay in the diagnosis of Trichomoniasis vaginalis. *Diagnostic microbiology and infectious disease* 2012;74(1):49-53.
34. Benzaken A, Galban E, Antunes W, et al. Diagnosis of gonococcal infection in high risk women using a rapid test. *Sexually transmitted infections* 2006;82(suppl 5):v26-v28.
35. Vallely AJ, Pomat WS, Homer C, et al. Point-of-care testing and treatment of sexually transmitted infections to improve birth outcomes in high-burden, low-income settings: Study protocol for a cluster randomized crossover trial (the WANTAIM Trial, Papua New Guinea). *Wellcome Open Research* 2019;4
36. Saweri O, Batura N, Al Adawiyah R, et al. The cost and cost-effectiveness of point-of-care testing and treatment for sexually transmitted and genital Infections in pregnancy in low- and middle-income countries. International Health Economics Assocation Congress. Basel, 2019.
37. Kuznik A, Lamorde M, Nyabigambo A, et al. Antenatal syphilis screening using point-of-care testing in Sub-Saharan African countries: a cost-effectiveness analysis. *PLoS medicine* 2013;10(11)
38. Rydzak CE, Goldie SJ. Cost-effectiveness of rapid point-of-care prenatal syphilis screening in sub-Saharan Africa. *Sexually transmitted diseases* 2008;35(9):775-84.
39. Vickerman P, Peeling R, Terris-Prestholt F, et al. Modelling the cost-effectiveness of introducing rapid syphilis tests into an antenatal syphilis screening programme in Mwanza, Tanzania. *Sexually transmitted infections* 2006;82(suppl 5):v38-v43.
40. Blandford JM, Gift TL, Vasaikar S, et al. Cost-effectiveness of on-site antenatal screening to prevent congenital syphilis in rural eastern Cape Province, Republic of South Africa. *Sexually transmitted diseases* 2007;34(7):S61-S66.
41. Romoren M, Hussein F, Steen TW, et al. Costs and health consequences of chlamydia management strategies among pregnant women in sub-Saharan Africa. *Sexually transmitted infections* 2007;83(7):558-66.
42. Larson BA, Lembela-Bwalya D, Bonawitz R, et al. Finding a needle in the haystack: the costs and cost-effectiveness of syphilis diagnosis and treatment during pregnancy to prevent congenital syphilis in Kalomo District of Zambia. *PLoS One* 2014;9(12)
43. Shelley KD, Ansbro ÉM, Ncube AT, et al. Scaling down to scale up: a health economic analysis of integrating point-of-care syphilis testing into antenatal care in Zambia during pilot and national rollout implementation. *PloS one* 2015;10(5)
44. Owusu-Edusei Jr K, Gift TL, Ballard RC. Cost-effectiveness of a dual non-treponemal/treponemal syphilis point-of-care test to prevent adverse pregnancy outcomes in sub-Saharan Africa. *Sexually transmitted diseases* 2011;38(11):997-1003.
45. Sweeney S, Mosha JF, Terris-Prestholt F, et al. The costs of accessible quality assured syphilis diagnostics: informing quality systems for rapid syphilis tests in a Tanzanian setting. *Health policy and planning* 2014;29(5):633-41.
46. Bristow CC, Larson E, Anderson LJ, et al. Cost-effectiveness of HIV and syphilis antenatal screening: a modelling study. *Sex Transm Infect* 2016;92(5):340-46.
47. Mallma P, Garcia P, Carcamo C, et al. Rapid syphilis testing is cost-effective even in low-prevalence settings: the CISNE-PERU experience. *PloS one* 2016;11(3)
48. Obure CD, Gaitan-Duarte H, Saenz RL, et al. A comparative analysis of costs of single and dual rapid HIV and syphilis diagnostics: results from a randomised controlled trial in Colombia. *Sexually transmitted infections* 2017;93(7):482-86.

For peer review only

49. Levin CE, Steele M, Atherly D, et al. Analysis of the operational costs of using rapid syphilis tests for the detection of maternal syphilis in Bolivia and Mozambique. *Sexually transmitted diseases* 2007;34(7):S47-S54.
50. Terris-Prestholt F, Vickerman P, Torres-Rueda S, et al. The cost-effectiveness of 10 antenatal syphilis screening and treatment approaches in Peru, Tanzania, and Zambia. *International Journal of Gynecology & Obstetrics* 2015;130:S73-S80.
51. Kuznik A, Muhumuza C, Komakech H, et al. Antenatal syphilis screening using point-of-care testing in low-and middle-income countries in Asia and Latin America: a cost-effectiveness analysis. *PLoS One* 2015;10(5)
52. Kuznik A, Lamorde M, Nyabigambo A, et al. Antenatal syphilis screening using point-of-care testing in Sub-Saharan African countries: a cost-effectiveness analysis. *PLoS Med* 2013;10(11):e1001545.
53. Shelley KD, Ansbro ÉM, Ncube AT, et al. Scaling down to scale up: a health economic analysis of integrating point-of-care syphilis testing into antenatal care in Zambia during pilot and national rollout implementation. *PloS one* 2015;10(5):e0125675.
54. Kuznik A, Muhumuza C, Komakech H, et al. Antenatal syphilis screening using point-of-care testing in low-and middle-income countries in Asia and Latin America: a cost-effectiveness analysis. *PLoS One* 2015;10(5):e0127379.
55. Larson BA, Lembela-Bwalya D, Bonawitz R, et al. Finding a needle in the haystack: the costs and cost-effectiveness of syphilis diagnosis and treatment during pregnancy to prevent congenital syphilis in Kalomo District of Zambia. *PLoS One* 2014;9(12):e113868.
56. Wang H, Liddell CA, Coates MM, et al. Global, regional, and national levels of neonatal, infant, and under-5 mortality during 1990–2013: a systematic analysis for the Global Burden of Disease Study 2013. *The Lancet* 2014;384(9947):957-79.
57. Kassebaum NJ, Bertozzi-Villa A, Coggeshall MS, et al. Global, regional, and national levels and causes of maternal mortality during 1990–2013: a systematic analysis for the Global Burden of Disease Study 2013. *The Lancet* 2014;384(9947):980-1004.
58. Mola G, Kirby B. Discrepancies between national maternal mortality data and international estimates: the experience of Papua New Guinea. *Reproductive health matters* 2013;21(42):191-202.
59. Mola G. Manual of standard managements in obstetrics and gynaecology for doctors. *HEOs and nurses in Papua New Guinea Sixth Edition ed* 2010
60. Government of Papua New Guinea. National Health Plan 2011–2020: National Department of Health, Government of Papua New Guinea, 2010.
61. Hou X, Khan MM, Pulford J, et al. Service delivery by health facilities in Papua New Guinea: report based on a countrywide health facility survey: The World Bank, 2018.
62. World Health Organization. Papua New Guinea-WHO Country Cooperation Strategy 2016-2020: Manila: WHO Regional Office for the Western Pacific, 2016.
63. World Health Organization. The world health report 2006: working together for health: World Health Organization 2006.
64. Hou XK, M. Mahmud; Pulford, Justin; Saweri, Olga; Demir, Ibrahim; Haider, Rifat; Ahmed, Shakil;. Service delivery by health facilities in Papua New Guinea : report based on a countrywide health facility survey (English). : The WorldBank Group, 2018.
65. Vassall A, Sweeney S, Kahn J, et al. Reference case for estimating the costs of global health services and interventions. 2017
66. Husereau D, Drummond M, Petrou S, et al. Consolidated health economic evaluation reporting standards (CHEERS)—explanation and elaboration: a report of the ISPOR health economic evaluation publication guidelines good reporting practices task force. *Value in Health* 2013;16(2):231-50.

For peer review only

67. Drummond MF, Sculpher MJ, Claxton K, et al. Methods for the economic evaluation of health care programmes: Oxford university press 2015.
68. Batura N, Pulkki-Brännström A-M, Agrawal P, et al. Collecting and analysing cost data for complex public health trials: reflections on practice. *Global health action* 2014;7(1):23257.
69. Mogyorosy Z, Smith P. The main methodological issues in costing health care services: a literature review. York, UK: Centre for Health Economics, 2005.
70. Wiseman V, Mangham LJ, Cundill B, et al. A cost-effectiveness analysis of provider interventions to improve health worker practice in providing treatment for uncomplicated malaria in Cameroon: a study protocol for a randomized controlled trial. *Trials* 2012;13(1):4.
71. Wiseman V, Ogochukwu E, Emmanuel N, et al. A cost-effectiveness analysis of provider and community interventions to improve the treatment of uncomplicated malaria in Nigeria: study protocol for a randomized controlled trial. *Trials* 2012;13(1):81.
72. Conteh L, Walker D. Cost and unit cost calculations using step-down accounting. *Health policy and planning* 2004;19(2):127-35.
73. Nixon RM, Wonderling D, Grieve RD. Non-parametric methods for cost-effectiveness analysis: the central limit theorem and the bootstrap compared. *Health economics* 2010;19(3):316-33.
74. Fenwick E, Claxton K, Sculpher M. Representing uncertainty: the role of cost-effectiveness acceptability curves. *Health economics* 2001;10(8):779-87.
75. Verguet S, Murphy S, Anderson B, et al. Public finance of rotavirus vaccination in India and Ethiopia: an extended cost-effectiveness analysis. *Vaccine* 2013;31(42):4902-10.
76. Verguet S, Kim JJ, Jamison DT. Extended Cost-Effectiveness Analysis for Health Policy Assessment: A Tutorial. *PharmacoEconomics* 2016;34(9):913-23. doi: 10.1007/s40273-016-0414-z
77. Bourguignon F, Chakravarty SR. The measurement of multidimensional poverty. Poverty, Social Exclusion and Stochastic Dominance: Springer 2019:83-107.
78. Alkire S, Roche JM, Ballon P, et al. Multidimensional poverty measurement and analysis: Oxford University Press, USA 2015.
79. Honeycutt AA, Khavjou OA, Jones DJ, et al. Helping the noncompliant child: An assessment of program costs and cost-effectiveness. *Journal of child and family studies* 2015;24(2):499-504.
80. Tandon A, Cashin C. Assessing public expenditure on health from a fiscal space perspective. Washington DC, USA: World Bank, 2010.
81. Heller PS. The prospects of creating 'fiscal space'for the health sector. *Health Policy and Planning* 2006;21(2):75-79.
82. Briggs A. Probabilistic analysis of cost-effectiveness models: statistical representation of parameter uncertainty. *Value in Health* 2005;8(1):1-2.

For peer review only

Authors' contributions: VW, NB, and OPMS led the design of the economic evaluation. All authors contributed to the conceptualisation, funding acquisition, methodology and formal analysis of the WANTAIM trial within which this study is nested. NB, OPMS, VW, AV, WP, CM, and MR contributed to data collection and acquisition for the economic evaluation. NB was responsible for the initial drafting of this manuscript, and all authors contributed to the review of this manuscript and provided comments. All authors read and approved the final manuscript.

Funding statement: This study is funded by the Joint Global Health Trials scheme (UK Department for International Development, Medical Research Council, Wellcome Trust; MR/N006089/1); National Health and Medical Research Council, Australia (Project Grant 1084429); and Swiss National Science Foundation Research for Development award (IZ07Z0_160909/1)

Competing interests statement: The authors have no competing interests.

Patient consent: Not required.

For peer review only

Protocol for an economic evaluation alongside a cluster-randomised trial of point-of-care testing and treatment of sexually transmitted and genital infections during pregnancy in Papua New Guinea (WANTAIM trial)

Authors

Batura N, Saweri OPM, Vallely AJ, Pomat WS, Homer C, Guy R, Luchters S, Mola GDL, Vallely LM, Morgan C, Kariwiga G, Wand H, Rogerson S, Tabrizi S, Whiley DM, Low N, Peeling R, Siba P, Riddell MA, Laman M, Bolnga J, Robinson L, Morewaya J, Badman SG, Kelly-Hanku A, Toliman P, Peter W, Peach E, Garland SM, Kaldor JM, and Wiseman V

Batura N (corresponding author)
Institute for Global Health
University College London
30 Guilford Street
London WC1 1EH
United Kingdom
Email: n.batura@ucl.ac.uk

Saweri OPM
The Kirby Institute, University of New South Wales
Papua New Guinea Institute of Medical Research
Email: nsaweri@kirby.unsw.edu.au

Vallely AJ
The Kirby Institute, University of New South Wales
Papua New Guinea Institute of Medical Research
Email: avallely@kirby.unsw.edu.au

Pomat WS
Papua New Guinea Institute of Medical Research
Email: william.pomat@pngimr.org.pg

Homer C
The Burnet Institute
Email: caroline.homer@burnet.edu.au

Guy R
The Kirby Institute, University of New South Wales
Email: Rguy@kirby.unsw.edu.au

Luchters S
The Burnet Institute
Email: stanley.luchters@burnet.edu.au

Mola GDL
School of Medicine and Health Sciences, University of Papua New Guinea
Email: glenmola@dg.com.pg

Vallely LM
The Kirby Institute, University of New South Wales
Email: lvallely@kirby.unsw.edu.au

Morgan C,
The Burnet Institute
Email: chris.morgan@burnet.edu.au

For peer review only

Kariwiga G
Milne Bay Provincial Health Authority, Papua New Guinea
Email: gkariwiga6@gmail.com

Wand H
The Kirby Institute, University of New South Wales
Email: hwand@kirby.unsw.edu.au

Rogerson S
University of Melbourne,
Email: sroger@unimelb.edu.au

Tabrizi S
The Royal Women's Hospital Melbourne
Email: Sepehr.Tabrizi@thewomens.org.au

Whiley DM
University of Queensland
Email: d.whiley@uq.edu.au

Low N
University of Bern
Email: nicola.low@ispm.unibe.ch

Peeling R
London School of Hygiene and Tropical Medicine
Email: rosanna.peeling@lshtm.ac.uk

Siba P
Papua New Guinea Institute of Medical Research
Email: peter.siba@pngimr.org.pg

Riddell MA
The Kirby Institute, University of New South Wales
Email: mriddell@kirby.unsw.edu.au

Laman M
Papua New Guinea Institute of Medical Research
Email: drmlaman@yahoo.com

Bolnga J
Papua New Guinea Institute of Medical Research
Email: johnbolnga@gmail.com

Robinson L
Papua New Guinea Institute of Medical Research
The Burnet Institute
Email: leanne.robinson@burnet.edu.au

Morewaya J
Milne Bay Provincial Health Authority, Papua New Guinea
Email: jacob.morewaya@yahoo.com.au

Badman SG
The Kirby Institute, University of New South Wales
Email: sbadman@kirby.unsw.edu.au

For peer review only

Kelly-Hanku A
The Kirby Institute, University of New South Wales
Papua New Guinea Institute of Medical Research
Email: a.kelly@unsw.edu.au

Toliman P
Papua New Guinea Institute of Medical Research
Email: pamelatoliman@gmail.com

Peter W
Provincial Health Office, Madang, Papua New Guinea
Email: wilfredpeterisalive777@gmail.com

Peach E
The Burnet Institute
Email: liz.peach@burnet.edu.au

Garland SM
the Royal Women's Hospital Melbourne
Email: Suzanne.Garland@thewomens.org.au

Kaldor JM
The Kirby Institute, University of New South Wales
Email: jkaldor@kirby.unsw.edu.au

Wiseman V
The Kirby Institute, University of New South Wales
Email: Virginia.Wiseman@lshtm.ac.uk

For peer review only

BMJ Open

Protocol for an economic evaluation alongside a cluster-randomised trial of point-of-care testing and treatment of sexually transmitted and genital infections during pregnancy in Papua New Guinea (WANTAIM trial)

Journal:	*BMJ Open*
Manuscript ID	bmjopen-2020-046308.R1
Article Type:	Protocol
Date Submitted by the Author:	29-Mar-2021
Complete List of Authors:	Batura, Neha; University College London, Institute for Global Health<br>saweri, olga; University of New South Wales, The Kirby Institute; Papua New Guinea Institute of Medical Research, Population Health and Demography<br>Vallely, Andrew; Papua New Guinea Institute of Medical Research, Sexual and Reproductive Health Unit; UNSW Sydney, The Kirby Institute<br>Pomat, Willie; PNG Institute of Medical Research, Infection and Immunity<br>Homer, Caroline ; Burnet Institute; University of Technology Sydney, Faculty of Health<br>Guy, Rebecca; The Kirby Instute, University of New South Wales, Sexual Health Program<br>Luchters, Stanley; Burnet Institute, Centre for International Health; Universiteit Gent<br>Mola, Glen ; School of Medicine and Health Sciences, University of Papua New Guinea<br>Vallely, Lisa; UNSW Australia, Kirby Institute -Faculty of Medicine<br>Morgan, Christopher; Burnet Institute,<br>Kariwiga, Grace; Milne Bay Provincial Health Authority<br>Wand, Handan; The Kirby Institute for Infection and Immunity in Society, University of New South Wales<br>Rogerson, Stephen; University of Melbourne, Doherty Institute, Department of Medicine<br>Tabrizi, Sepehr; The Royal Women’s Hospital,<br>Whiley, David; The University of Queensland, Queensland Children's Medical Research Institute<br>Low, Nicola; University of Bern, Bern, Switzerland, Institute of Social and Preventive Medicine<br>Peeling, Rosanna; London School of Hygiene and Tropical Medicine, Clinical Research<br>Siba, Peter; The Papua New Guinea Institute of Medical Research, Sexual and Reproductive Health Unit<br>Riddell, Michaela; University of New South Wales Faculty of Science, Kirby Institute<br>Laman, M.; Papua New Guinea Institute of Medical Research,<br>Bolnga, John ; Papua New Guinea Institute of Medical Research; The Kirby Institute for infection and immunity in society<br>Robinson, Leanne; Papua New Guinea Institute of Medical Research; Burnet Institute

	Morewaya, Jacob; Milne Bay Provincial Health Authority<br>Badman, Steven; UNSW Australia, Kirby Insitute<br>Kelly-Hanku, Angela; Papua New Guinea Institute of Medical Research; The Kirby Institute<br>Toliman, Pamela ; Papua New Guinea Institute of Medical Research<br>Peter, Wilfred; Provincial Health Office<br>Peach, Elizabeth; Burnet Institute<br>Garland, Suzanne; The Royal Women's Hospital, Microbiology and Infectious Diseases Department<br>Kaldor, John; University of New South Wales, The Kirby Institute<br>Wiseman, Virginia; The Kirby Institute; London School of Hygiene & Tropical Medicine
**Primary Subject Heading**:	Health economics
Secondary Subject Heading:	Global health, Epidemiology, Health services research
Keywords:	HEALTH ECONOMICS, Public health < INFECTIOUS DISEASES, Protocols & guidelines < HEALTH SERVICES ADMINISTRATION & MANAGEMENT

SCHOLARONE™
Manuscripts

I, the Submitting Author has the right to grant and does grant on behalf of all authors of the Work (as defined in the below author licence), an exclusive licence and/or a non-exclusive licence for contributions from authors who are: i) UK Crown employees; ii) where BMJ has agreed a CC-BY licence shall apply, and/or iii) in accordance with the terms applicable for US Federal Government officers or employees acting as part of their official duties; on a worldwide, perpetual, irrevocable, royalty-free basis to BMJ Publishing Group Ltd ("BMJ") its licensees and where the relevant Journal is co-owned by BMJ to the co-owners of the Journal, to publish the Work in this journal and any other BMJ products and to exploit all rights, as set out in our licence.

The Submitting Author accepts and understands that any supply made under these terms is made by BMJ to the Submitting Author unless you are acting as an employee on behalf of your employer or a postgraduate student of an affiliated institution which is paying any applicable article publishing charge ("APC") for Open Access articles. Where the Submitting Author wishes to make the Work available on an Open Access basis (and intends to pay the relevant APC), the terms of reuse of such Open Access shall be governed by a Creative Commons licence – details of these licences and which Creative Commons licence will apply to this Work are set out in our licence referred to above.

Other than as permitted in any relevant BMJ Author's Self Archiving Policies, I confirm this Work has not been accepted for publication elsewhere, is not being considered for publication elsewhere and does not duplicate material already published. I confirm all authors consent to publication of this Work and authorise the granting of this licence.

For peer review only

Protocol for an economic evaluation alongside a cluster-randomised trial of point-of-care testing and treatment of sexually transmitted and genital infections during pregnancy in Papua New Guinea (WANTAIM trial)

Authors

Batura N, Saweri OPM, Vallely AJ, Pomat WS, Homer C, Guy R, Luchters S, Mola GDL, Vallely LM, Morgan C, Kariwiga G, Wand H, Rogerson S, Tabrizi S, Whiley DM, Low N, Peeling R, Siba P, Riddell MA, Laman M, Bolnga J, Robinson L, Morewaya J, Badman SG, Kelly-Hanku A, Toliman P, Peter W, Peach E, Garland SM, Kaldor JM, and Wiseman V

Batura N (corresponding author)
Institute for Global Health
University College London
30 Guilford Street
London WC1 1EH, UK
Email: n.batura@ucl.ac.uk

Saweri OPM
The Kirby Institute, University of New South Wales, Sydney, Australia
Papua New Guinea Institute of Medical Research, Goroka, Papua New Guinea
Email: nsaweri@kirby.unsw.edu.au

Vallely AJ
The Kirby Institute, University of New South Wales, Sydney, Australia
Papua New Guinea Institute of Medical Research, Goroka, Papua New Guinea
Email: avallely@kirby.unsw.edu.au

Pomat WS
Papua New Guinea Institute of Medical Research, Goroka, Papua New Guinea
Email: william.pomat@pngimr.org.pg

Homer C
The Burnet Institute, Melbourne, Australia
Email: caroline.homer@burnet.edu.au

Guy R
The Kirby Institute, University of New South Wales, Sydney, Australia
Email: Rguy@kirby.unsw.edu.au

Luchters S
The Burnet Institute, Melbourne, Australia
Email: stanley.luchters@burnet.edu.au

Mola GDL
School of Medicine and Health Sciences, University of Papua New Guineam, Port Moresby, Papua New Guinea
Email: glenmola@dg.com.pg

Vallely LM
The Kirby Institute, University of New South Wales, Sydney, Australia

For peer review only

Email: lvallely@kirby.unsw.edu.au

Morgan C
The Burnet Institute, Melbourne, Australia
Email: chris.morgan@burnet.edu.au

Kariwiga G
Milne Bay Provincial Health Authority, Alotau, Papua New Guinea
Email: gkariwiga6@gmail.com

Wand H
The Kirby Institute, University of New South Wales, Sydney, Australia
Email: hwand@kirby.unsw.edu.au

Rogerson S
University of Melbourne, Melbourne, Australia
Email: sroger@unimelb.edu.au

Tabrizi S
The Royal Women's Hospital Melbourne, Melbourne, Australia
Email: Sepehr.Tabrizi@thewomens.org.au

Whiley DM
University of Queensland, Brisbane, Australia
Email: d.whiley@uq.edu.au

Low N
University of Bern, Bern, Switzerland
Email: nicola.low@ispm.unibe.ch

Peeling R
London School of Hygiene and Tropical Medicine, London, UK
Email: rosanna.peeling@lshtm.ac.uk

Siba P
Papua New Guinea Institute of Medical Research, Goroka, Papua New Guinea
Email: peter.siba@pngimr.org.pg

Riddell MA
The Kirby Institute, University of New South Wales, Sydney, Australia
Email: mriddell@kirby.unsw.edu.au

Laman M
Papua New Guinea Institute of Medical Research, Goroka, Papua New Guinea
Email: drmlaman@yahoo.com

Bolnga J
Papua New Guinea Institute of Medical Research, Goroka, Papua New Guinea
Email: johnbolnga@gmail.com

Robinson L

For peer review only

Papua New Guinea Institute of Medical Research, Goroka, Papua New Guinea
The Burnet Institute, Melbourne, Australia
Email: leanne.robinson@burnet.edu.au

Morewaya J
Milne Bay Provincial Health Authority, Alotau, Papua New Guinea
Email: jacob.morewaya@yahoo.com.au

Badman SG
The Kirby Institute, University of New South Wales, Sydney, Australia
Email: sbadman@kirby.unsw.edu.au

Kelly-Hanku A
The Kirby Institute, University of New South Wales, Sydney, Australia
Papua New Guinea Institute of Medical Research, Goroka, Papua New Guinea
Email: a.kelly@unsw.edu.au

Toliman P
Papua New Guinea Institute of Medical Research, Goroka, Papua New Guinea
Email: pamelatoliman@gmail.com

Peter W
Provincial Health Office, Madang, Papua New Guinea
Email: wilfredpeterisalive777@gmail.com

Peach E
The Burnet Institute, Melbourne, Australia
Email: liz.peach@burnet.edu.au

Garland SM
The Royal Women's Hospital Melbourne, Melbourne, Australia
Email: Suzanne.Garland@thewomens.org.au

Kaldor JM
The Kirby Institute, University of New South Wales, Sydney, Australia
Email: jkaldor@kirby.unsw.edu.au

Wiseman V
The Kirby Institute, University of New South Wales, Sydney, Australia
London School of Hygiene and Tropical Medicine, London, UK
Email: Virginia.Wiseman@lshtm.ac.uk

Word count (excluding title page, abstract, tables, acknowledgements, contributions, references): 3431

For peer review only

Abstract

Introduction: Left untreated, sexually transmitted and genital infections (henceforth STIs) in pregnancy can lead to serious adverse outcomes for mother and child. Papua New Guinea (PNG) has amongst the highest prevalence of curable STIs including syphilis, chlamydia, gonorrhoea, trichomoniasis and bacterial vaginosis, and high neonatal mortality rates. Diagnosis and treatment of these STIs in PNG relies upon syndromic management. Advances in STI diagnostics through point-of-care (PoC) testing using GeneXpert technology holds promise for resource-constrained countries such as PNG. This paper describes the planned economic evaluation of a cluster-randomised crossover trial comparing antenatal PoC testing and immediate treatment of curable STIs with standard antenatal care in two provinces in PNG.

Methods and analysis: Cost-effectiveness of the PoC intervention compared with standard antenatal care will be assessed prospectively over the trial period (2017-2021) from societal and provider perspectives. Incremental cost-effectiveness ratios will be calculated for the primary health outcome, a composite measure of the proportion of either preterm birth and/or low birth weight; for life years saved; for disability-adjusted life years averted; and for non-health benefits (financial risk protection and improved health equity). Scenario analyses will be conducted to identify scale-up options, and budget impact analysis will be undertaken to understand short-term financial impacts of intervention adoption on the national budget. Deterministic and probabilistic sensitivity analysis will be conducted to account for uncertainty in key model inputs.

Ethics and dissemination: This study has ethical approval from the Institutional Review Board of the PNG Institute of Medical Research; the Medical Research Advisory Committee of the PNG National Department of Health; the Human Research Ethics Committee of the University of New South Wales; and the Research Ethics Committee of the London School of Hygiene and Tropical Medicine. Findings will be disseminated through national stakeholder meetings, conferences, peer-reviewed publications and policy briefs.

Trial registration: ISRCTN37134032

Keywords

Cost-effectiveness analysis, economic evaluation, budget impact, equity, sexually transmitted infections, point-of-care testing, Papua New Guinea

For peer review only

Strengths and limitations of this study

- This protocol will assist in designing economic evaluations for similar complex public health interventions, especially those that seek to capture both health and non-health impacts of point-of-care testing for STIs in low– and middle-income countries (LMICs).
- This protocol follows the Consolidated Health Economic Evaluation Reporting Standards, and guidelines from the Global Health Cost Consortium to design and report economic evaluations nested in a randomised controlled trial and will include individual level patient cost and health service use data.
- The planned budget impact and affordability analyses will contribute to the understanding of fiscal space for investments in maternal and child health at provincial and country levels.
- This analysis builds on conventional cost-effectiveness analysis by including non-health benefits such as financial risk protection that are key criteria for equitable resource allocation and the design of health benefit packages in many LMICs including PNG.
- The planned analyses for the base case scenario adopt only a 12-month time horizon but we propose to model the costs and benefits over the lifetime of mother and their babies using published data.

For peer review only

INTRODUCTION

In 2017, it was estimated that every day, globally, more than 1 million people acquire any of the four common curable sexually transmitted infections: chlamydia, gonorrhoea, syphilis, and trichomoniasis [1-3]. Left untreated, sexually transmitted and genital infections such as bacterial vaginosis (henceforth, referred to as STIs) are associated with adverse pregnancy and birth outcomes including spontaneous abortion, miscarriage, stillbirth, pre-term birth, low birth weight, postpartum endometritis, premature rupture of membranes, and various sequelae in newborn infants owing to mother-to-child transmission such as ophthalmia neonatorum [4-13].

In Papua New Guinea (PNG), prevalence of STIs is high in the general population and among pregnant women [14]. Clinical studies in PNG show that around 50% of all pregnant women test positive for one or more STIs at their first antenatal care (ANC) visit [15 16], with gonorrhoea, chlamydia, trichomoniasis, and bacterial vaginosis most commonly diagnosed [15-18]. There is evidence from resource constrained settings to suggest that increased screening for HIV and syphilis in pregnancy is correlated with a reduction in perinatal and infant morbidity and mortality [19 20]. In this high burden and low resource setting, poor access to ANC leads to missed opportunities for early diagnosis and clinical intervention [21].

Traditional STI diagnosis for infections other than HIV and syphilis relies on microscopy, culture, and/or serology that requires technical resources and expertise that may not be readily available in all low- and middle-income country (LMIC) settings [22 23]. The long waiting period for results also deters some people from returning to collect their results [24]. In settings where laboratory services are not available, syndromic management, which relies on clinical presentation, is most often used to inform treatment decisions. This strategy fails to accurately identify causative pathogens or detect asymptomatic infections and consequently leads to negligible impact on health outcomes [24 25]. The development of accurate rapid diagnostic tests for HIV and syphilis used at point-of-care (PoC) has improved their detection, testing coverage and the number of patients accurately diagnosed and treated [26-28]. From an equity standpoint, PoC testing has been shown to improve access to testing and treatment particularly amongst remote and hard to reach populations [26 29]. However, the success of HIV and syphilis PoC diagnosis is yet to be replicated for other common curable STIs, including chlamydia and gonorrhoea [30-34].

For peer review only

The Women and Newborn Trial of Antenatal Interventions and Management (WANTAIM) study is the first randomised trial to evaluate the effectiveness and cost-effectiveness of PoC STI testing and treatment to improve birth outcomes in high-burden settings (Trial registration number: ISRCTN37134032) [35]. This paper aims to describe the rationale and methodological approach for the economic evaluation component of this large-scale trial involving 4,600 pregnant women in PNG.

In recent years, the evidence base for the cost and cost-effectiveness of PoC testing for STIs in pregnancy has grown, including in LMICs. A recent systematic review [36] identified that the bulk of these studies were conducted in Africa or Latin and South America [37-52], with no studies undertaken in East Asia or the Pacific. Most of the studies investigated the cost and cost-effectiveness of PoC testing for syphilis in pregnancy compared to no screening, syndromic management or onsite laboratory testing. Only one study evaluated testing for HIV and testing and treatment for syphilis [48], another for chlamydia [41], and none evaluated testing for gonorrhoea, trichomoniasis, or bacterial vaginosis. Few studies evaluated the costs and cost-effectiveness of the test and treatment package combined. Despite widespread acknowledgement of the high out-of-pocket costs incurred by women and their families in accessing testing and treatment in many LMICs, the studies in the review were largely conducted from the provider perspective [40 41 45 49 50 52-55]. Further, none of the studies presented estimates of affordability or budget impact and none analysed non-health related outcomes such as equity or financial risk protection [36].

STUDY SETTING

Papua New Guinea

In PNG, pregnant women and their infants experience a high burden of adverse health outcomes with PNG recording one of the highest maternal mortality ratios and neonatal mortality rates in the world: 584 per 100,000 and 25 per 1000 live births, respectively, compared with global figures of 209 and 18 [56 57]. In 2012, 20% of births in PNG were preterm birth and/or low birth weight, both key contributors to neonatal mortality [58].

Pregnant women in PNG experience a high burden of curable STIs. Findings from a country-wide bio-behavioural survey of STIs in pregnancy indicated that the prevalence of chlamydia was 23%, gonorrhoea 14%, and trichomoniasis 22%, with 44% of women having at least one of these infections [5]. Another study evaluating the feasibility of a novel PoC testing and treatment strategy for STIs in PNG found that 54% of women had one or more of chlamydia,

For peer review only

gonorrhoea, trichomoniasis, or bacterial vaginosis, and the prevalence of each of these STIs was 19%, 11%, 38%, and 18% respectively [22]. Similar prevalence rates of STIs were observed in a study of malaria prevention in pregnancy [6]. In these studies, between 65-80% of infections among pregnant women were asymptomatic indicating the need for more accurate diagnosis at PoC.

In PNG, national guidelines for ANC state that PoC testing and treatment for HIV and syphilis should be undertaken for all pregnant women at the first ANC clinic visit. For women who test positive, treatment according to national guidelines is offered along with partner testing [59]. However, despite the high prevalence of chlamydia, gonorrhoea, trichomoniasis and bacterial vaginosis amongst pregnant women, detection and treatment rely on syndromic management according to national guidelines [59].

Health services in Papua New Guinea

In PNG, health services are organized into seven levels of care. Levels 1-4 offer primary care at community aid posts, sub-health centres, health centres, and rural/district hospitals. The majority of Level 1-3 facilities are managed and staffed by health extension officers (HEOs), Nursing Officers (NOs), midwives and community health workers (CHW); Level 4 facilities i.e., rural/district hospitals usually have a doctor on staff. Population coverage varies from about 5,000 to 20,000 per facility and the average distance travelled to reach a facility is 7-8 km. Secondary level care is provided at provincial/regional/national referral hospitals (Levels 5-7), which cover an average population of 200,000 and 300,000 in one or more provinces [60 61]. Health workforce distribution is suboptimal, with 0.5 physicians per 10,000 population [62], compared to the World Health Organization (WHO) recommended ratio of 10 physicians per 10,000 population [63]. Healthcare is predominantly provided by public health facilities that are either financed and operated by the PNG government or by churches with financial support from the government [64].

The WANTAIM trial

WANTAIM aims to test the effectiveness of antenatal PoC testing and treatment for STIs to improve maternal and newborn outcomes in PNG. WANTAIM is being implemented in two provinces in PNG – Madang and East New Britain. Data collection continues despite the challenges of the COVID-19 pandemic, and the trial is due to finish in late 2021 [35].

WANTAIM is a cluster-randomised crossover trial and the unit of randomization is a primary

For peer review only

health care centre and its catchment area. Ten geographically distinct clusters have been assigned in a 1:1 ratio to intervention and control arms in the first phase of the trial. The end of the first phase of the trial is followed by a short washout period of two to three months, at the end of which each cluster will cross over to participate in the alternative trial arm in the second phase. The study participants are women attending their first ANC visit, aged over 16 years and less than 26 weeks gestation (assessed by ultrasound) (n=4,600). Newborn infants are followed up within 72 hours of birth.

Pregnant women recruited into the study receive routine ANC as per PNG national guidelines including sulphadoxine-pyrimethamine (SP) for malaria prevention; iron and folate supplementation; tetanus toxoid immunization; HIV and syphilis screening (and treatment if required). Women in the control arm receive STI syndromic management if they report symptoms of a genital infection (abdominal pain, discharge). They also provide a urine sample for diagnostic testing on GeneXpert in the study laboratory. If positive for STI at their last test, they receive treatment during the first postnatal visit. Women in the intervention arm of the trial provide a self-collected vaginal specimen for PoC STI testing, and same-day treatment as necessary, at the following time points:

- At enrolment (<26 weeks gestation)
- One month after trial enrolment; and
- At 34-36 weeks antenatal follow-up.

The primary outcome of the trial is a composite measure of two events, the proportion of women and their newborn infants in each trial arm who experience either a preterm birth (<37 weeks gestation) and/or low birth weight (<2500g).

The study has ethical approval from the Institutional Review Board of the Papua New Guinea (PNG) Institute of Medical Research (IRB number 1608); the Medical Research Advisory Committee of the PNG National Department of Health (MRAC number 16.24); the Human Research Ethics Committee of the University of New South Wales (HREC number 16708); and the Research Ethics Committee of the London School of Hygiene and Tropical Medicine (REC number 12009).

A full description of the WANTAIM intervention and trial design is described elsewhere [35]. The purpose of this paper is to fully describe the methods for the economic evaluation of the trial.

For peer review only

AIMS AND OBJECTIVES

The economic evaluation aims to assess the cost-effectiveness and affordability of PoC testing and treatment of curable STIs in pregnancy compared to standard care from a provider and societal perspective. The specific objectives of the economic evaluation are to:

1. Estimate total financial and economic costs of the PoC STI intervention;
2. Model incremental cost-effectiveness of the intervention compared to standard care;
3. Extend the incremental cost-effectiveness analysis to include equity related measures of impact; and
4. Conduct a budget impact analysis to assess the affordability of implementing the intervention at the national level or in target areas/populations.

These planned analyses will adhere to the Consolidated Health Economic Evaluation Reporting Standards and established guidelines from the Global Health Cost Consortium for conducting and reporting economic evaluation for global health trials [65 66].

METHODS: ECONOMIC EVALUATION OF THE WANTAIM TRIAL

Costing data

Cost data collection is guided by the perspective adopted for the economic evaluation. For WANTAIM, direct and indirect costs will be collected from the provider perspective and societal perspective – the latter including any costs incurred by pregnant women and their families. The different cost categories and data sources are summarised in Table 1. A combination of top-down and bottom-up costing approaches will be used [65] and the time horizon for the main trial-based economic evaluation will be 12 months.

Table 1: Cost category, and data sources

Description	Type of cost	Data sources	Sample size
Provider costs			
Costs of implementing WANTAIM	Direct	Project accounts of implementing agencies	N/A
Cost of providing ANC services	Direct	Health facilities	10 health facilities
Cost of increased workload of facility staff associated with PoC testing and treatment	Indirect	Patient pathway and health worker observation data collected as part of the health facility assessment	20-30
Participant costs			
Costs of care seeking	Direct and indirect	Participant case report forms	4,600

For peer review only

Provider costs are incurred by the institutions implementing the PoC testing intervention across the start-up, implementation and monitoring phases of the trial. The cost data will be sourced from financial records, programme documents and consultation with project staff. A step-down costing methodology will be used whereby costs from project accounts are entered into a customized tool created in Microsoft Excel, which is adapted each year to reflect the changing cost structure of the trial during the start-up and implementation phases.

Financial costs will be converted to economic costs i.e. any donated goods or volunteer time that do not appear in the programme accounting data will be added to the cost sheets and assigned a current market value [67 68]. Key informant interviews with programme leads will assist in identifying donated or subsidised items and in allocating joint costs between programme components. The allocation of joint staff costs is informed by monthly staff timesheets. Research costs will not be included in the cost-effectiveness analysis. However, start-up costs will be reported and differentiated from implementation costs to enable decision-makers to gauge the costs associated with the initial activities and expenditures necessary to develop PoC testing and integration with standard ANC [69].

Provider (treatment) costs are incurred by Provincial Health Authorities, who manage ANC, delivery, and postnatal visits, and Church Health Services; non-state providers who access a mix of government and institutional funds. Primary data on the average unit cost of care will be collected from all health facilities participating in the WANTAIM trial. A simple cost-capture form has been developed for facility data collection adapted from other costing studies led by members of this team [70 71]. Data from this form will be used to complement existing data from centre reports, patients' records and published national reports relating to ANC, labour and birth care, and PNC visits. Costs of services provided will also be calculated using a step-down approach. [72]

Participant (treatment) costs are the direct and indirect costs of health care seeking incurred by women and their families such as medical costs, transport costs, and the opportunity costs in terms of lost productivity due to care seeking visits. These will be estimated for standard treatment episodes in the control arms and for treatment episodes in the intervention arm to gauge changes in out-of-pocket costs of care-seeking and time dedicated to care-seeking for participants. Data on the direct and indirect costs incurred by participants are being collected

For peer review only

from all trial participants (n=4600) in both arms of the trial at enrolment and three follow up visits through participant case report forms (CRFs). The participant cost data will be summed and analysed as cross-sectional data to gauge the economic burden borne by participants and their households that is alleviated due to PoC testing and treatment of STIs in pregnancy.

Health service use

Health service utilization for all trial participants in the intervention and control arms will be estimated using data collected via a take-home aide memoire and participant CRFs. The aide memoire is provided to all participants at recruitment, who use this tool to make notes about the facility visits that they make or attend between the WANTAIM follow-up visits. The aide memoire also allows them to make notes about any costs associated with those visits. At the WANTAIM follow-up visits, these notes serve as prompts for questions about service utilization and costs of care-seeking, which are recorded in the CRFs.

Proposed analyses

Cost and cost-effectiveness analyses:

A base case analysis will be undertaken alongside the trial to estimate the cost-effectiveness of the intervention compared with standard care as implemented. The base case will include all start-up costs and implementation costs. Costs will be presented in current prices in PNG Kina and International Dollars. All costs will be adjusted for inflation using the Consumer Price Index (CPI) for PNG and will be converted to 2021 International Dollars (INT$) using the 2021 Purchasing Power Parity (PPP) conversion factor for PNG. Costs and outcomes will be converted to present values using an annual discount rate of 3% in the base-case, and annual rates of 0%, 6% and 9% in sensitivity analyses.

For base case analysis, results will be presented in terms of total financial and economic costs of the intervention and incremental cost-effectiveness ratios (ICERs) for the primary outcome i.e. the proportion of women and their newborns who experience either preterm birth and/or low birth weight. ICERs will be calculated as the arithmetic mean difference in cost between the intervention and control arms, divided by the arithmetic mean difference in effect. To maximise comparability with other trials, ICERs will also be reported in terms of cost per life year saved and cost per disability adjusted life year (DALY) averted (see 'Modelling' section for details).

A descriptive analysis of missing data will be undertaken to inform the base-case assumption

For peer review only

regarding the missing data mechanism (the probability that missing data are independent or not on the observed or unobserved values). Appropriate methods will be used to handle the missing data, which may include mean imputation, multiple imputations, available case analysis, inverse probability weighting, or likelihood methods [73]. Sensitivity analysis will be conducted as appropriate.

The data on costs and outcomes for the period of trial follow-up will be at the individual level, allowing evaluation of uncertainty of the cost-effectiveness estimates using non-parametric bootstrapping [74]. Cost-effectiveness acceptability curves (CACs) will be generated to further describe uncertainty around the cost estimates [75]. CACs indicate the proportion of the estimates produced by bootstrapping that would be 'acceptable' below a range of willingness to pay thresholds, where willingness to pay is the value placed on an additional pregnant woman appropriately tested for STIs in pregnancy. Sensitivity analyses will take into account the uncertainty in key parameters that may have been affected by the COVID-19 pandemic, such as staff or drugs costs.

Modelling:

The base case analysis will have a time horizon of up to 12 months. If the intervention demonstrates clinical effectiveness over that period, we will employ a cohort decision analytic model to examine the cost-effectiveness of the intervention over a newborn's lifetime. A Markov model will be used to estimate the long-term health benefits, healthcare costs and cost-effectiveness of the PoC intervention compared to standard care, drawing on results of the WANTAIM trial and available published data. The point of entry into the model will be 'tested for STIs'. There are two possible states for women: infected or uninfected. Women identified as infected and then treated may recover and stay healthy, become re-infected or die. Health outcomes will therefore depend on treatment compliance and include live birth without infection (healthy infant), live birth with infection, preterm and/ or low birth weight, and neonatal death. The model will be used to project differences between the intervention and control arms in life years saved, DALYs averted, and lifetime healthcare costs. Sensitivity analyses will also be conducted within this model.

Equity impact of the intervention:

The equity impact of the intervention will be investigated by conducting an extended cost-effectiveness analysis (E-CEA). The E-CEA broadens the scope of the CEA by incorporating

For peer review only

health equity and financial protection considerations for the most vulnerable sections of the population, that are likely to have the highest need [76]. This will be done across three domains: by exploring improving health gains, with particular reference to the poorest socio-economic group; reduction in the out-of-pocket expenses faced by households seeking care; and improved financial protection or reducing the number of households that sink into poverty due to catastrophic health spending [77]. For the E-CEA, provider and participant cost data (Table 1) will be synthesised with data on service utilization that will be collected via participant CRFs that are completed at enrolment into the trial and at three follow up trial visits. All results will be presented by socio-economic quintiles. Given that socio-economic groups may not differ greatly within clusters, a multidimensional poverty index (MDPI) will be derived from socio-economic and income data collected from all trial participants at enrolment. The use of an MDPI provides a more nuanced understanding of socioeconomic status of households as it takes monetary and non-monetary dimensions of deprivation into account [78]. This enables the differentiation between population groups who may all be relatively poor in monetary dimensions such as income or asset ownership [79] Thus, the consideration of other non-monetary attributes (e.g. housing) allows us to distinguish between households that are homogenously asset or cash poor in this study setting [79].

Scale-up and budget impact analysis:

The costs and cost-effectiveness of the intervention will also be considered in a scale-up scenario, in which any start-up costs will be excluded as they are considered sunk costs [80]. The budget impact of the scale up scenario will be explored by an analysis of fiscal space for programme delivery using a generalised fiscal space assessment method [81 82] and probabilistic analyses to determine a set of cost-effectiveness thresholds [75 83].

Patient and public involvement

WANTAIM trial participants were involved in providing data for the study.

DISCUSSION

To our knowledge, this paper is the first protocol for the economic evaluation of PoC STI testing and treatment in pregnancy in an LMIC setting. The proposed analyses aim to assess the cost-effectiveness of the intervention as well as its affordability and equity impact. The analyses will adhere to international guidelines for conducting and reporting economic evaluation studies and provide transparency in how they are conducted. The findings of the

For peer review only

economic evaluation will provide decision-makers in PNG and similar settings evidence on the relative value for money of this intervention and the likely level of investment required for implementation at scale. The findings of this study will be disseminated through national stakeholder meetings, conferences, peer-reviewed publications and policy briefs.

DISSEMINATION

The findings of the economic evaluation of the WANTAIM trial will be disseminated to academic and policy-making communities, and the wider public, in a peer-reviewed journals and presented at relevant conferences in Papua New Guinea and globally.

For peer review only

Authors' contributions: VW, NB, and OPMS led the design of the economic evaluation. NB, OPMS, VW, AV, WP, CM, and MR contributed to data collection and acquisition for the economic evaluation. NB, OPMS, VW, will lead the formal data analysis for the economic evaluation. AV, WP, CH, RG, SL, GM, LV, CM, GK, HW, SR, ST, DW, NL, RP, PS, MR, ML, JB, LR, JM, SG, AKH, PT, WP, EP, SG, JK, and VW are the research team involved in conceptualisation, funding acquisition, methodology, data curation and/or formal analysis of the WANTAIM trial within which this economic evaluation is nested, and from where the outcome data for the economic evaluation will be used . NB was responsible for the initial drafting of this manuscript. All authors contributed substantially to the review of this manuscript and provided critical and constructive comments. All authors read and approved the final manuscript. NB, OPMS, VW, AV, LV, MR and SB contributed to responding to reviewer comments and the revision of the manuscript.

Acknowledgement: The study team would like to thank the WANTAIM trial participants, health facility staff and the provincial officers in PNG.

Funding statement: This study is funded by the Joint Global Health Trials scheme (UK Department for International Development, Medical Research Council, Wellcome Trust; MR/N006089/1); National Health and Medical Research Council, Australia (Project Grant 1084429); and Swiss National Science Foundation Research for Development award (IZ07Z0_160909/1)

Competing interests statement: The authors have no competing interests.

Patient consent: All eligible participants provided written informed consent to participate in the study and provide individual level data.

For peer review only

REFERENCES

1. Newman L, Rowley J, Vander Hoorn S, et al. Global estimates of the prevalence and incidence of four curable sexually transmitted infections in 2012 based on systematic review and global reporting. *PloS one* 2015;10(12):e0143304.
2. World Health Organization. Sexually transmitted infections: implementing the global STI strategy: World Health Organization, 2017.
3. Unemo M, Bradshaw CS, Hocking JS, et al. Sexually transmitted infections: challenges ahead. *The Lancet infectious diseases* 2017;17(8):e235-e79.
4. Mullick S, Watson-Jones D, Beksinska M, et al. Sexually transmitted infections in pregnancy: prevalence, impact on pregnancy outcomes, and approach to treatment in developing countries. *Sexually transmitted infections* 2005;81(4):294-302.
5. Vallely LM, Toliman P, Ryan C, et al. Prevalence and risk factors of Chlamydia trachomatis, Neisseria gonorrhoeae, Trichomonas vaginalis and other

sexually transmissible infections among women attending antenatal clinics in three provinces in Papua New Guinea: a cross-sectional survey. *Sexual health* 2016;13(5):420-27.
6. Wangnapi R, Soso S, Unger H, et al. Prevalence and risk factors for Chlamydia trachomatis, Neisseria gonorrhoeae and Trichomonas vaginalis infection in pregnant women in Papua New Guinea. *Sex Transm Infect* 2015;91(3):194-200.
7. Gravett MG, Nelson HP, DeRouen T, et al. Independent associations of bacterial vaginosis and Chlamydia trachomatis infection with adverse pregnancy outcome. *Jama* 1986;256(14):1899-903.
8. John Hopkins Study Investigators. Association of Chlamydia trachomatis and Mycoplasma hominis with intrauterine growth retardation and preterm delivery. The John Hopkins Study of Cervicitis and Adverse Pregnancy Outcome. *Am J Epidemiol* 1989;129(6):1247-57.
9. Elliott B, Brunham RC, Laga M, et al. Maternal gonococcal infection as a preventable risk factor for low birth weight. *Journal of infectious diseases* 1990;161(3):531-36.
10. de Attayde MJPM, Florêncio GLD, Gabiatti JRE, et al. Perinatal morbidity and mortality associated with chlamydial infection: a meta-analysis study. *The Brazilian Journal of Infectious Diseases* 2011;15(6):533-39.
11. Rours GIJ, Duijts L, Moll HA, et al. Chlamydia trachomatis infection during pregnancy associated with preterm delivery: a population-based prospective cohort study. *European journal of epidemiology* 2011;26(6):493-502.
12. Johnson HL, Ghanem KG, Zenilman JM, et al. Sexually transmitted infections and adverse pregnancy outcomes among women attending inner city public sexually transmitted diseases clinics. *Sexually transmitted diseases* 2011;38(3):167-71.
13. Kahn JG, Jiwani A, Gomez GB, et al. The cost and cost-effectiveness of scaling up screening and treatment of syphilis in pregnancy: a model. *PLoS One* 2014;9(1)
14. Blencowe H, Cousens S, Jassir FB, et al. National, regional, and worldwide estimates of stillbirth rates in 2015, with trends from 2000: a systematic analysis. *The Lancet Global Health* 2016;4(2):e98-e108.
15. Badman SG, Vallely LM, Toliman P, et al. A novel point-of-care testing strategy for sexually transmitted infections among pregnant women in high-burden settings: results of a feasibility study in Papua New Guinea. *BMC infectious diseases* 2016;16:250-50. doi: 10.1186/s12879-016-1573-4
16. Wangnapi RA, Soso S, Unger HW, et al. Prevalence and risk factors for Chlamydia trachomatis, Neisseria gonorrhoeae and Trichomonas vaginalis infection in pregnant women in Papua New Guinea. *Sexually Transmitted Infections* 2015;91(3):194-200. doi: 10.1136/sextrans-2014-051670
17. Vallely A, Page A, Dias S, et al. The prevalence of sexually transmitted infections in Papua New Guinea: a systematic review and meta-analysis. *PloS one* 2010;5(12):e15586-e86. doi: 10.1371/journal.pone.0015586
18. Vallely LM, Toliman P, Ryan C, et al. Prevalence and risk factors of Chlamydia trachomatis, Neisseria gonorrhoeae, Trichomonas vaginalis and other sexually transmissible infections among women attending antenatal

For peer review only

clinics in three provinces in Papua New Guinea: a cross-sectional survey. *Sexual Health* 2016;13(5):420-27. doi: https://doi.org/10.1071/SH15227
19. Watson-Jones D, Oliff M, Terris-Prestholt F, et al. Antenatal syphilis screening in sub-Saharan Africa: lessons learned from Tanzania. *Tropical Medicine & International Health* 2005;10(9):934-43. doi: 10.1111/j.1365-3156.2005.01473.x
20. Blencowe H, Cousens S, Kamb M, et al. Lives Saved Tool supplement detection and treatment of syphilis in pregnancy to reduce syphilis related stillbirths and neonatal mortality. *BMC public health* 2011;11(S3):S9.
21. Ministerial Task Force on Maternal Health in Papua New Guinea. Report of the Ministerial Task Force on Maternal Health in Papua New Guinea. . Port Moresby, Papua New Guinea: National Department of Health, Government of Papua New Guinea, 2009.
22. Badman SG, Vallely LM, Toliman P, et al. A novel point-of-care testing strategy for sexually transmitted infections among pregnant women in high-burden settings: results of a feasibility study in Papua New Guinea. *BMC infectious diseases* 2016;16(1):250.
23. Toskin I, Blondeel K, Peeling RW, et al. Advancing point of care diagnostics for the control and prevention of STIs: the way forward. *Sex Transm Infect* 2017;93(S4):S81-S88.
24. Vallely LM, Toliman P, Ryan C, et al. Performance of syndromic management for the detection and treatment of genital Chlamydia trachomatis, Neisseria gonorrhoeae and Trichomonas vaginalis among women attending antenatal, well woman and sexual health clinics in Papua New Guinea: a cross-sectional study. *BMJ open* 2017;7(12):e018630-e30. doi: 10.1136/bmjopen-2017-018630
25. Peeling RW. Testing for sexually transmitted infections: a brave new world? *Sexually Transmitted Infections* 2006;82(6):425-30. doi: 10.1136/sti.2005.017251
26. Mabey DC, Sollis KA, Kelly HA, et al. Point-of-Care Tests to Strengthen Health Systems and Save Newborn Lives: The Case of Syphilis. *PLOS Medicine* 2012;9(6):e1001233. doi: 10.1371/journal.pmed.1001233
27. Marks M, Mabey DC. The introduction of syphilis point of care tests in resource limited settings. *Expert review of molecular diagnostics* 2017;17(4):321-25. doi: 10.1080/14737159.2017.1303379 [published Online First: 2017/03/14]
28. Swartzendruber A, Steiner RJ, Adler MR, et al. Introduction of rapid syphilis testing in antenatal care: A systematic review of the impact on HIV and syphilis testing uptake and coverage. *International Journal of Gynaecology & Obstetrics* 2015;130 Suppl 1:S15-21.
29. Phang Romero Casas C, Martyn-St James M, Hamilton J, et al. Rapid diagnostic test for antenatal syphilis screening in low-income and middle-income countries: a systematic review and meta-analysis. *BMJ Open* 2018;8(2):e018132.
30. Nuñez-Forero L, Moyano-Ariza L, Gaitán-Duarte H, et al. Diagnostic accuracy of rapid tests for sexually transmitted infections in symptomatic women. *Sexually transmitted infections* 2016;92(1):24-28.

For peer review only

31. Pearce DM, Styles DN, Hardick JP, et al. A new rapid molecular point-of-care assay for Trichomonas vaginalis: preliminary performance data. *Sexually transmitted infections* 2013;89(6):495-97.
32. Sabido M, Hernandez G, Gonzalez V, et al. Clinic-based evaluation of a rapid point-of-care test for detection of Chlamydia trachomatis in specimens from sex workers in Escuintla, Guatemala. *Journal of clinical microbiology* 2009;47(2):475-76.
33. Hegazy MM, El-Tantawy NL, Soliman MM, et al. Performance of rapid immunochromatographic assay in the diagnosis of Trichomoniasis vaginalis. *Diagnostic microbiology and infectious disease* 2012;74(1):49-53.
34. Benzaken A, Galban E, Antunes W, et al. Diagnosis of gonococcal infection in high risk women using a rapid test. *Sexually transmitted infections* 2006;82(suppl 5):v26-v28.
35. Vallely AJ, Pomat WS, Homer C, et al. Point-of-care testing and treatment of sexually transmitted infections to improve birth outcomes in high-burden, low-income settings: Study protocol for a cluster randomized crossover trial (the WANTAIM Trial, Papua New Guinea). *Wellcome Open Research* 2019;4
36. Saweri O, Batura N, Al Adawiyah R, et al. The cost and cost-effectiveness of point-of-care testing and treatment for sexually transmitted and genital Infections in pregnancy in low- and middle-income countries. International Health Economics Assocation Congress. Basel, 2019.
37. Kuznik A, Lamorde M, Nyabigambo A, et al. Antenatal syphilis screening using point-of-care testing in Sub-Saharan African countries: a cost-effectiveness analysis. *PLoS medicine* 2013;10(11)
38. Rydzak CE, Goldie SJ. Cost-effectiveness of rapid point-of-care prenatal syphilis screening in sub-Saharan Africa. *Sexually transmitted diseases* 2008;35(9):775-84.
39. Vickerman P, Peeling R, Terris-Prestholt F, et al. Modelling the cost-effectiveness of introducing rapid syphilis tests into an antenatal syphilis screening programme in Mwanza, Tanzania. *Sexually transmitted infections* 2006;82(suppl 5):v38-v43.
40. Blandford JM, Gift TL, Vasaikar S, et al. Cost-effectiveness of on-site antenatal screening to prevent congenital syphilis in rural eastern Cape Province, Republic of South Africa. *Sexually transmitted diseases* 2007;34(7):S61-S66.
41. Romoren M, Hussein F, Steen TW, et al. Costs and health consequences of chlamydia management strategies among pregnant women in sub-Saharan Africa. *Sexually transmitted infections* 2007;83(7):558-66.
42. Larson BA, Lembela-Bwalya D, Bonawitz R, et al. Finding a needle in the haystack: the costs and cost-effectiveness of syphilis diagnosis and treatment during pregnancy to prevent congenital syphilis in Kalomo District of Zambia. *PLoS One* 2014;9(12)
43. Shelley KD, Ansbro ÉM, Ncube AT, et al. Scaling down to scale up: a health economic analysis of integrating point-of-care syphilis testing into antenatal care in Zambia during pilot and national rollout implementation. *PloS one* 2015;10(5)

For peer review only

44. Owusu-Edusei Jr K, Gift TL, Ballard RC. Cost-effectiveness of a dual non-treponemal/treponemal syphilis point-of-care test to prevent adverse pregnancy outcomes in sub-Saharan Africa. *Sexually transmitted diseases* 2011;38(11):997-1003.
45. Sweeney S, Mosha JF, Terris-Prestholt F, et al. The costs of accessible quality assured syphilis diagnostics: informing quality systems for rapid syphilis tests in a Tanzanian setting. *Health policy and planning* 2014;29(5):633-41.
46. Bristow CC, Larson E, Anderson LJ, et al. Cost-effectiveness of HIV and syphilis antenatal screening: a modelling study. *Sex Transm Infect* 2016;92(5):340-46.
47. Mallma P, Garcia P, Carcamo C, et al. Rapid syphilis testing is cost-effective even in low-prevalence settings: the CISNE-PERU experience. *PloS one* 2016;11(3)
48. Obure CD, Gaitan-Duarte H, Saenz RL, et al. A comparative analysis of costs of single and dual rapid HIV and syphilis diagnostics: results from a randomised controlled trial in Colombia. *Sexually transmitted infections* 2017;93(7):482-86.
49. Levin CE, Steele M, Atherly D, et al. Analysis of the operational costs of using rapid syphilis tests for the detection of maternal syphilis in Bolivia and Mozambique. *Sexually transmitted diseases* 2007;34(7):S47-S54.
50. Terris‐Prestholt F, Vickerman P, Torres‐Rueda S, et al. The cost‐effectiveness of 10 antenatal syphilis screening and treatment approaches in Peru, Tanzania, and Zambia. *International Journal of Gynecology & Obstetrics* 2015;130:S73-S80.
51. Kuznik A, Muhumuza C, Komakech H, et al. Antenatal syphilis screening using point-of-care testing in low-and middle-income countries in Asia and Latin America: a cost-effectiveness analysis. *PLoS One* 2015;10(5)
52. Kuznik A, Lamorde M, Nyabigambo A, et al. Antenatal syphilis screening using point-of-care testing in Sub-Saharan African countries: a cost-effectiveness analysis. *PLoS Med* 2013;10(11):e1001545.
53. Shelley KD, Ansbro ÉM, Ncube AT, et al. Scaling down to scale up: a health economic analysis of integrating point-of-care syphilis testing into antenatal care in Zambia during pilot and national rollout implementation. *PloS one* 2015;10(5):e0125675.
54. Kuznik A, Muhumuza C, Komakech H, et al. Antenatal syphilis screening using point-of-care testing in low-and middle-income countries in Asia and Latin America: a cost-effectiveness analysis. *PLoS One* 2015;10(5):e0127379.
55. Larson BA, Lembela-Bwalya D, Bonawitz R, et al. Finding a needle in the haystack: the costs and cost-effectiveness of syphilis diagnosis and treatment during pregnancy to prevent congenital syphilis in Kalomo District of Zambia. *PLoS One* 2014;9(12):e113868.
56. Wang H, Liddell CA, Coates MM, et al. Global, regional, and national levels of neonatal, infant, and under-5 mortality during 1990–2013: a systematic analysis for the Global Burden of Disease Study 2013. *The Lancet* 2014;384(9947):957-79.
57. Kassebaum NJ, Bertozzi-Villa A, Coggeshall MS, et al. Global, regional, and national levels and causes of maternal mortality during 1990–2013: a

For peer review only - http://bmjopen.bmj.com/site/about/guidelines.xhtml

systematic analysis for the Global Burden of Disease Study 2013. *The Lancet* 2014;384(9947):980-1004.
58. Mola G, Kirby B. Discrepancies between national maternal mortality data and international estimates: the experience of Papua New Guinea. *Reproductive health matters* 2013;21(42):191-202.
59. Mola G. Manual of standard managements in obstetrics and gynaecology for doctors. *HEOs and nurses in Papua New Guinea Sixth Edition ed* 2010
60. Government of Papua New Guinea. National Health Plan 2011–2020: National Department of Health, Government of Papua New Guinea, 2010.
61. Hou X, Khan MM, Pulford J, et al. Service delivery by health facilities in Papua New Guinea: report based on a countrywide health facility survey: The World Bank, 2018.
62. World Health Organization. Papua New Guinea-WHO Country Cooperation Strategy 2016-2020: Manila: WHO Regional Office for the Western Pacific, 2016.
63. World Health Organization. The world health report 2006: working together for health: World Health Organization 2006.
64. Hou XK, M. Mahmud; Pulford, Justin; Saweri, Olga; Demir, Ibrahim; Haider, Rifat; Ahmed, Shakil;. Service delivery by health facilities in Papua New Guinea : report based on a countrywide health facility survey (English). : The WorldBank Group, 2018.
65. Vassall A, Sweeney S, Kahn J, et al. Reference case for estimating the costs of global health services and interventions. 2017
66. Husereau D, Drummond M, Petrou S, et al. Consolidated health economic evaluation reporting standards (CHEERS)—explanation and elaboration: a report of the ISPOR health economic evaluation publication guidelines good reporting practices task force. *Value in Health* 2013;16(2):231-50.
67. Drummond MF, Sculpher MJ, Claxton K, et al. Methods for the economic evaluation of health care programmes: Oxford university press 2015.
68. Batura N, Pulkki-Brännström A-M, Agrawal P, et al. Collecting and analysing cost data for complex public health trials: reflections on practice. *Global health action* 2014;7(1):23257.
69. Mogyorosy Z, Smith P. The main methodological issues in costing health care services: a literature review. York, UK: Centre for Health Economics, 2005.
70. Wiseman V, Mangham LJ, Cundill B, et al. A cost-effectiveness analysis of provider interventions to improve health worker practice in providing treatment for uncomplicated malaria in Cameroon: a study protocol for a randomized controlled trial. *Trials* 2012;13(1):4.
71. Wiseman V, Ogochukwu E, Emmanuel N, et al. A cost-effectiveness analysis of provider and community interventions to improve the treatment of uncomplicated malaria in Nigeria: study protocol for a randomized controlled trial. *Trials* 2012;13(1):81.
72. Conteh L, Walker D. Cost and unit cost calculations using step-down accounting. *Health policy and planning* 2004;19(2):127-35.
73. Faria R, Gomes M, Epstein D, et al. A guide to handling missing data in cost-effectiveness analysis conducted within randomised controlled trials. *Pharmacoeconomics* 2014;32(12):1157-70.

For peer review only

74. Nixon RM, Wonderling D, Grieve RD. Non‐parametric methods for cost‐effectiveness analysis: the central limit theorem and the bootstrap compared. *Health economics* 2010;19(3):316-33.
75. Fenwick E, Claxton K, Sculpher M. Representing uncertainty: the role of cost‐effectiveness acceptability curves. *Health economics* 2001;10(8):779-87.
76. Verguet S, Murphy S, Anderson B, et al. Public finance of rotavirus vaccination in India and Ethiopia: an extended cost-effectiveness analysis. *Vaccine* 2013;31(42):4902-10.
77. Verguet S, Kim JJ, Jamison DT. Extended Cost-Effectiveness Analysis for Health Policy Assessment: A Tutorial. *PharmacoEconomics* 2016;34(9):913-23. doi: 10.1007/s40273-016-0414-z
78. Bourguignon F, Chakravarty SR. The measurement of multidimensional poverty. Poverty, Social Exclusion and Stochastic Dominance: Springer 2019:83-107.
79. Alkire S, Roche JM, Ballon P, et al. Multidimensional poverty measurement and analysis: Oxford University Press, USA 2015.
80. Honeycutt AA, Khavjou OA, Jones DJ, et al. Helping the noncompliant child: An assessment of program costs and cost-effectiveness. *Journal of child and family studies* 2015;24(2):499-504.
81. Tandon A, Cashin C. Assessing public expenditure on health from a fiscal space perspective. Washington DC, USA: World Bank, 2010.
82. Heller PS. The prospects of creating 'fiscal space'for the health sector. *Health Policy and Planning* 2006;21(2):75-79.
83. Briggs A. Probabilistic analysis of cost‐effectiveness models: statistical representation of parameter uncertainty. *Value in Health* 2005;8(1):1-2.

For peer review only
